# Efficiency Improvement and SEM Visualization of Enzymatic Hydrolysis-Based Valorization of Oat Hulls

**DOI:** 10.3390/ijms27167148

**Published:** 2026-08-10

**Authors:** Ekaterina I. Kashcheyeva, Vera V. Budaeva

**Affiliations:** Bioconversion Laboratory, Institute for Problems of Chemical and Energetic Technologies, Siberian Branch of the Russian Academy of Sciences (IPCET SB RAS), Biysk 659322, Russia

**Keywords:** waste valorization, oat hulls, pretreatment, hydrolysis, enzyme cocktails

## Abstract

The utilization of agro-industrial waste in the bioeconomy is crucial for reducing the environmental impact. This study investigated the valorization of a common oat processing waste—oat hulls—using enzyme preparations of different origin and enzyme cocktails based on them. Oat hulls were used in native form and after chemical pretreatment. The following three enzyme preparations were used for enzymatic hydrolysis: Agrocell Plus, CelloLux-A, and Ultraflo Max. An enzyme cocktail consisting of the three enzyme preparations was found to provide a conversion degree of 45% for native oat hulls with simultaneous hydrolysis of cellulose and hemicelluloses, with glucose accounting for only 41% of the total reducing sugars. Enzymatic hydrolysis of the chemically pretreated product confirmed the effectiveness of using enzyme cocktails: the yield of reducing sugars increased 1.4–1.8 times compared to the individual enzymes. The use of the enzyme cocktail consisting of the three enzyme preparations provided a 99% conversion of the accessible fraction of the substrate. Visualization by scanning electron microscopy (SEM) of the hydrolysis of native oat hulls revealed the disappearance of substrate surface irregularities, while in the case of the chemically pretreated product, the substrate disintegration was observed. The comparison of the SEM results of the two substrates before and after hydrolysis confirms the effectiveness of the enzyme cocktails. The contribution of glucose to the total concentration of reducing sugars in the hydrolyzates obtained from the chemically pretreated oat hulls was 85–91%, which allows for predicting the successful application of these hydrolyzates as nutrient media for the biosynthesis of value-added products.

## 1. Introduction

Lignocellulosic biomass (LCB) is the most abundant and low-cost resource in the world [1,2]. LCB-derived products have great potential to replace those obtained from fossil fuels, thereby playing a key role in the transition to bioeconomy [3,4]. The conversion of LCB is crucial for the global carbon cycle, as it involves the transfer of a significant amount of carbon from its fixed reservoir into the atmosphere [5]. However, developing an efficient, cost-effective, and environmentally friendly process for the maximum conversion of LCB components into value-added products, which would allow for the complete utilization of biomass components while preventing the formation of undesirable by-products, remains a challenging task [6,7]. Enzymes offer a promising pathway for LCB conversion due to their high selectivity in the hydrolysis of polysaccharides (cellulose and hemicelluloses) into simple/fermentable sugars such as glucose and xylose [3,8,9]. A high conversion degree of a lignocellulosic substrate can be achieved by using efficient and robust enzymes with no their irreversible binding to lignin and end-product inhibition. However, harnessing the potential of LCB remains challenging due to several factors—physicochemical, structural, and compositional—that limit the conversion of natural polysaccharides [2,3].

Agro-industrial waste generated during the production and processing of grain crops has significant economic potential due to its low cost and high value [2,8,9,10,11]. When improperly managed, such as through incineration or landfilling, lignocellulosic waste has a negative environmental impact due to greenhouse gas emissions. According to available data, agro-industrial waste is the second largest source (19.9%) of greenhouse gas emissions [8]. The negative consequences of improper waste management can be mitigated through the implementation of efficient and environmentally friendly processing technologies to obtain monosaccharides suitable for further conversion into food, chemical, and fuel products [8,12,13]. Bioconversion has become an environmentally sustainable approach for producing value-added products, enabling waste reduction and decreasing dependence on non-renewable resources [8].

Oat (*Avena sativa*) is one of the most widely known cereal crops in the world, ranking seventh in global grain production. Oat processing generates large quantities of oat hulls, as the hull accounts for approximately 25% of the total seed mass. Oat hulls contain approximately 23% cellulose, 35% hemicelluloses, 25% lignin, and 3% starch, although their exact composition varies depending on growing conditions [8,14]. The composition of oat hulls makes them a promising feedstock for the production of value-added products [8,14], including biogenic silica [15], which can be isolated from cereal hulls via chemical or biotechnological route [15,16,17].

The efficient conversion of agro-industrial waste into value-added products is challenging due to the complex interplay of lignin, hemicellulose, and cellulose, rendering plant fibers highly resistant to hydrolysis [10,18,19]. Pretreatment, enzymatic hydrolysis, and sugar fermentation are the main stages in the conversion of lignocellulosic waste into monosaccharides and subsequent biosynthetic products. Among these, pretreatment and enzyme cost (or enzymatic hydrolysis cost) are considered the main cost drivers [11,20,21,22,23,24,25]. Commercial cellulase enzymes contain a range of hydrolytic enzymes, including cellulases, hemicellulases, endoglucanases, and lytic polysaccharide monooxygenases (LPMOs), which break down various bonds between functional groups of polymers contained in LCB. The synergistic action of enzymes enhances substrate hydrolysis [8,20]. Thus, the development of highly efficient and low-cost enzyme preparations for lignocellulose hydrolysis remains one of the major research platforms in the field of LCB conversion [1].

Making an enzyme preparation containing three cellulase components (endoglucanases, cellobiohydrolases, and β-glucosidases) in the correct proportion for complete hydrolysis of cellulosic substrates is a challenging task [20]. The use of multienzyme compositions for LCB hydrolysis is key to an efficient process. To achieve maximum substrate hydrolysis (>70%), a balanced composition of cellulolytic enzymes is required, which can increase the hydrolysis rate, reduce enzyme loading, and thus lower the overall cost of monosaccharide production [26,27]. Making efficient cellulolytic enzyme preparation requires more components than thought before. Selection of enzymes/proteins and chemicals for the preparation of cocktail requires preliminary knowledge about the efficiency of non-catalytic proteins and enzymes or other activators in real-time biomass hydrolysis. The simplest way to obtain a highly efficient multienzyme mixture is to combine two or three enzyme preparations of different origin (mainly fungi and bacteria), which vary in the quantity and type of cellulolytic enzymes they contain [20]. Such a mixture allows for the manifold increase in the degree of hydrolysis of the lignocellulosic substrate at a lower protein concentration or FPU level. As a result, the enzymatic hydrolysis process will only benefit both practically and economically. For instance, β-glucosidases from different sources display different substrate specificities for cellooligosaccharides and cellobiose: some β-glucosidases efficiently hydrolyze cellooligosaccharides with a degree of polymerization from 2 to 6, while others effectively hydrolyze only C2 sugars [21]. In some cases, non-cellulolytic enzymes also serve as excellent supplements, decreasing the required cellulase loading and accelerating biomass liquefaction at a high initial substrate concentration. The study [28] reported that the hydrolysis of sugarcane bagasse was increased by adding xylanases to cellulase, which reduced cellulase consumption by 20% when 20% of the cellulase was replaced with xylanases. Furthermore, xylanases increase the hydrolysis efficiency of both cellulose and hemicelluloses by disrupting the interaction between lignin and xylan and, consequently, removing the xylan coating [3].

The objectives of this work were to improve the efficiency of bioconversion of oat hulls before and after pretreatment using enzyme cocktails and to visualize the process by SEM.

## 2. Results and Discussion

### 2.1. Substrate Characteristics

The composition of native oat hulls (NOHs) and chemically pretreated oat hulls (CPOHs) used as substrates is presented in Table 1. It should be noted that the content of the main components of NOHs in this study falls within the ranges published previously [8]: 23–48% cellulose, 16–35% hemicelluloses, 16–25% lignin, and 2–10% ash. Despite the diversity of real industrial waste as oat hulls worldwide, a similar composition is observed for this specific, naturally calibrated lignocellulosic material. For these reasons, it can be stated that there are no issues with the enzymatic hydrolysis of chemically pretreated oat hulls in the factory environment because native wastes from different grain elevators are alike in composition [2].

After pretreatment, the cellulose content in CPOHs increased by 25.3% compared to the untreated material (66.1% vs. 40.8%). The increased cellulose content indicates that a portion of the cellulose located in the cell walls was released from the hemicellulose–lignin matrix following the pretreatment [19]. Pentosans (6.8% vs. 31.0%) were almost completely hydrolyzed during the pretreatment process, resulting in a 24.2% decrease in their mass content. At the same time, the lignin content in CPOHs did not decrease significantly, amounting to 17.1%, which is only 4.6% lower than in NOHs. The ash content increased from 5.5 wt% to 9.7 wt% due to the complete removal of pentosans, whereas the ash components retaining in the solid phase.

Pretreatment of oat hulls with nitric acid led to the removal of hemicelluloses and lignin, which are known to affect the resistance of cellulose to enzymatic hydrolysis in lignocellulosic biomass, as well as to limit the enzyme activity during the hydrolysis process [2,7,8]. In addition, the action of dilute nitric acid on lignin in oat hulls induces profound chemical changes, specifically simultaneous oxidation and condensation, accompanied by partial deactivation of this polymer with respect to enzymes [29]. This phenomenon is consistent with the fundamental understanding of the action of dilute acids on cereal husks [8]: lignin is redistributed into a condensed structure and hemicellulose is depolymerized whereby cellulose becomes more accessible to enzymes. These observations suggest that this pretreatment method will promote efficient enzymatic hydrolysis of the resulting substrate.

### 2.2. Enzymatic Hydrolysis

#### 2.2.1. Enzymatic Hydrolysis of Oat Hulls

The characteristics of the hydrolyzates derived from NOHs after 72 h hydrolysis are presented in Table 2.

The efficiency assessment of individual enzymes during the hydrolysis of NOHs (experiments #1–3) demonstrates that only 13–28% of the accessible fraction of the substrate is converted. With Agrocell Plus, the RS yield was 1.7 times higher than that obtained with Cellolux-A and 2.1 times higher than that with Ultraflo Max. Agrocell Plus is characterized by the highest cellulase and β-glucanase activities described below (see Section 3.2), which confirms the obtained results (an RS yield of 20% on a substrate weight basis).

The use of enzyme cocktails for the hydrolysis of NOHs led to a 1.2–5.0-fold increase in the substrate conversion degree: in experiments #6 and 7, the RS yield reached maximum values of 34–45%. The addition of Ultraflo Max to Agrocell Plus or Cellolux-A (experiments #4 and 5) did not significantly increase the RS yield (2%). In contrast, mixing two enzyme preparations containing the same enzymes (experiment #6) but of different origins (Agrocell Plus and Cellolux-A) resulted in a 1.7–2.8-fold increase in the substrate conversion degree compared to individual enzymes. The substrate hydrolysis rate in the presence of lignin is known to depend on the type of enzyme used [12]. In this context, cellulase is the least effective compared to xylanase and glucosidases. This statement is consistent with the results obtained. When Agrocell Plus (a cellulase activity of 4000 U/g and a xylanase activity of 400 U/g) was mixed with Cellolux-A (a cellulase activity of 2000 U/g and a xylanase activity of 6000 U/g), the RS yield increased from 12 to 20% to 34%.

The cocktail consisting of all three enzyme preparations (experiment #7) showed the highest hydrolysis efficiency as follows: the substrate conversion degree increased by 25–36% compared to the individual preparations, thereby reaching 45%. Mixing three enzyme preparations of different origin yielded an enzyme cocktail that provided maximum hydrolysis of NOHs, with the conversion degree of the accessible substrate fraction reaching 63%. The contribution of glucose to the total RS concentration was 41–48%, indicating simultaneous hydrolysis of cellulose and hemicelluloses.

The obtained results of enzymatic hydrolysis of NOHs are explained by their composition (high hemicellulose and lignin contents), as well as by the well-known resistance of oat hulls to any biological impact, since the hulls naturally ensure the preservation of the grain during ripening and subsequent storage [8,18]. The multilayered structure of the cell wall, the intermolecular and intramolecular hydrogen bonds, and the strong hydrophobicity of cellulose are known to determine the natural recalcitrance of lignocellulosic biomass, increasing its resistance to enzymatic cleavage and hindering the efficient hydrolysis of cellulose into fermentable sugars [12,30]. The lignin–carbohydrate complex, with its high contents of pentosans (31.0%) and lignin (22.7%) in NOHs, also acts as a barrier to enzymes. Lignin irreversibly adsorbs enzymes, reducing their activity and hydrolysis efficiency. It should be noted that the achieved RS yield on a substrate weight basis in Experiment #7 exceeds the previously reported results by a factor of 3.7 (12.2% in [31]), which is due to the exceptionally higher efficiency of the cocktail of three enzyme preparations acting on NOHs. This once again confirms the improvement in enzymatic hydrolysis efficiency of native (untreated) lignocellulose using enzyme cocktails. This observation is consistent with the general understanding of enzyme synergy in cocktails [20].

#### 2.2.2. Enzymatic Hydrolysis of Chemically Pretreated Oat Hulls (CPOHs)

The feedstock was pretreated in order to enhance the accessibility of the substrate to enzymes [19]. It is well known that pretreatment methods can effectively disrupt the recalcitrant structure of lignocellulose, thereby facilitating subsequent enzymatic hydrolysis and the conversion of the feedstock into fermentable sugars and other biosynthetic products [8,18,24]. As a result of NOH pretreatment, significant removal of hemicelluloses and lignin occurred, leading to the disruption of the lignin–hemicellulose matrix, a reduction in the number of lignin binding sites for enzymes, and loosening of the biomass structure. These alterations in substrate properties suggest an acceleration of the hydrolysis process for CPOHs. The results of enzymatic hydrolysis of CPOHs are summarized in Table 3. Comparison of the individual enzyme preparations (experiments #1–3) revealed that the maximum substrate conversion degree was achieved with Agrocell Plus and Ultraflo Max: the RS yield in both cases was 1.3-fold higher than with Cellolux-A. Agrocell Plus is characterized by the highest activities (Table 4), which accounts for the obtained results (an RS yield of 53% on a substrate weight basis). An unexpectedly high substrate conversion degree of CPOHs was demonstrated with Ultraflo Max (an RS yield of 51% on a substrate weight basis), since this enzyme preparation is positioned as a β-glucanase. This is explained by the fact that, in addition to the enzymes declared in its product datasheet (β-glucanase and xylanase), this enzyme preparation also possesses a sufficiently high cellulase activity. The use of individual enzymes results in the hydrolysis of the accessible fraction of the substrate (cellulose and pentosans) by 54–72%.

Mixing Agrocell Plus with Cellolux-A or Ultraflo Max led to hydrolysis of the accessible substrate fraction in experiments #4 and 6, corresponding to an RS yield of 71% on a substrate weight basis. This indicates that the enzymatic system of Agrocell Plus is the primary one, while the other two enzymes enhance its action. Similar phenomena have been described previously [3,8,20]. The maximum RS yield (72% on a substrate weight basis) was observed during enzymatic hydrolysis with three mixed enzyme preparations (experiment #7). Mixing the three enzyme preparations of different origin (experiment #7) yielded an enzyme cocktail that hydrolyzed the entire accessible fraction of the substrate: the RS yield on a hydrolyzables content basis was 99%. This is explained by the fact that enzyme cocktails containing the same enzymes in their formulation but obtained from different species (*Penicillium verruculosum* is a microbial producer in Agrocell Plus, *Trichoderma viride* (*reesei*) in CelloLux-A, and *Aspergillus oryzae* + *Trichoderma reesei* in Ultraflo Max) work synergistically. These preparations enhance each other’s positive characteristics and thereby increase hydrolysis efficiency [8,20,21]. According to [32], the use of enzyme cocktails with an excellent catalytic activity is the only solution to ensure stability in practical biocatalysis. In all cases, the high yields of reducing sugars on a hydrolyzables content basis confirm that the elevated ash content of 9.7% in the substrate had no effect on the hydrolysis efficiency.

Preparations derived from *Trichoderma* can often be found in the literature devoted to enzyme systems rich in exoglucanase. *Aspergillus* is often found in studies related to the synthesis of endoglucanase, whereas *Penicillium* is characterized as an excellent source of β-glucosidase. This complementary distribution of enzymatic capabilities explains why mixed fungal cultures and enzyme cocktails combining species from different genera have attracted increasing attention in recent years. Some studies have demonstrated that combinations of *Trichoderma* + *Aspergillus* + *Penicillium* can create more balanced cellulolytic systems than those obtained from individual strains [33]. These preparations derived from different sources complement each other’s enzymatic capabilities, enhance each other’s positive characteristics, and thereby enhance the hydrolysis efficiency [8,18,19]. According to [34], the use of enzyme cocktails with an excellent catalytic activity is the only solution to ensure stability in practical biocatalysis.

The ratio of glucose to xylose concentrations characterizes the obtained hydrolyzates as glucose-rich as follows: the contribution of glucose to the total RS concentration was 85–91%, while the contribution of xyloses was 9–12%. It should be noted that the contribution of glucose to the total RS concentration increased 1.9–2.1-fold compared to that in the hydrolysis of NOHs (41–48%), due to changes in the substrate composition after pretreatment. Since the cellulose content in CPOHs increased 1.6-fold relative to NOHs, while the pentosan content decreased 4.6-fold, these changes resulted in a glucose-to-xylose ratio with a predominant content of glucose. The production of predominantly glucose-rich hydrolyzates from lignocellulose establishes enzymatic hydrolysis with subsequent fermentation as a cornerstone technology that links waste reduction, innovations in economically demanded products, and the global agenda of sustainable development.

### 2.3. SEM Visualization of Enzymatic Hydrolysis of Two Substrates

**Figure 1:**
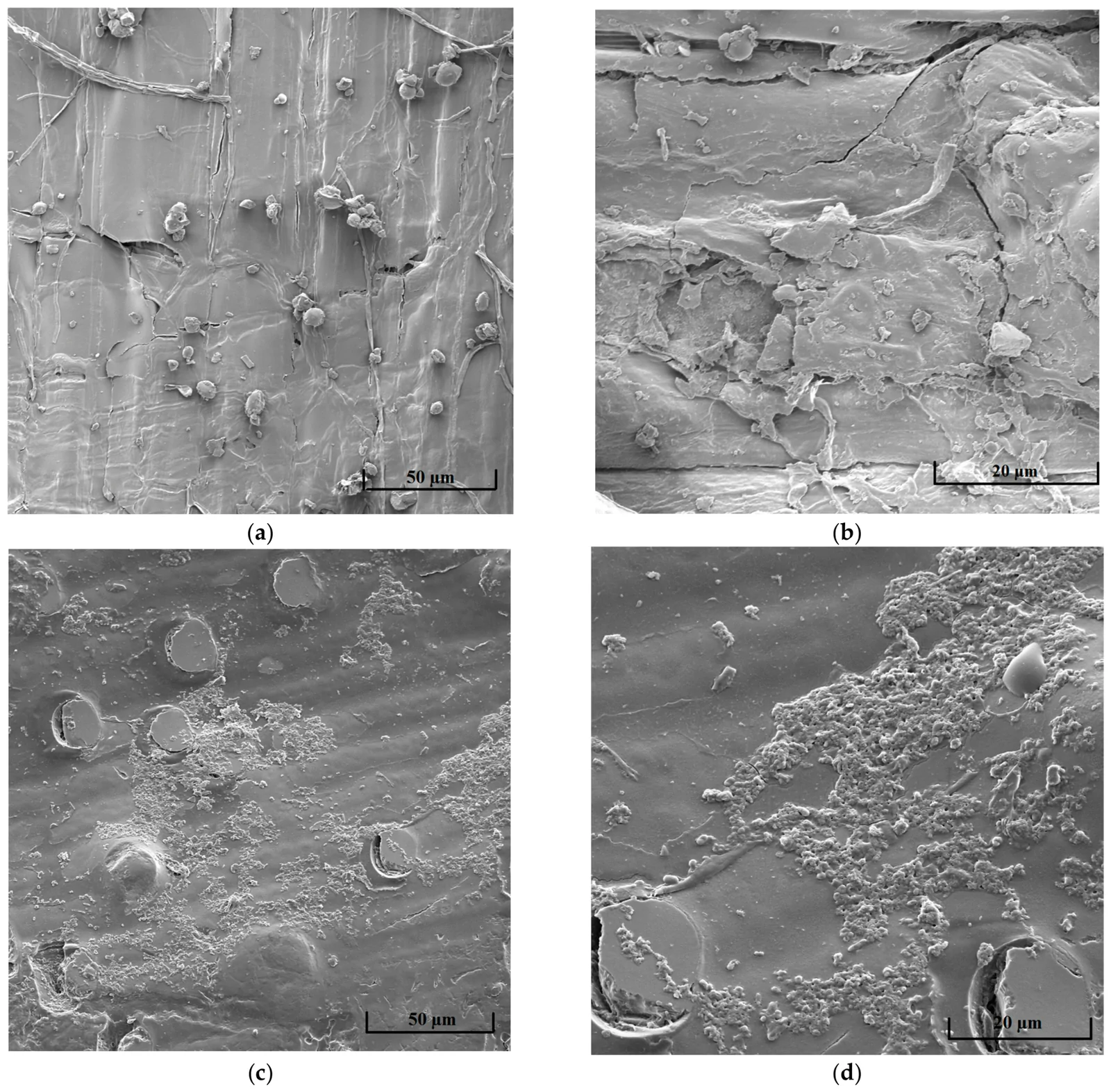
SEM visualization of enzymatic hydrolysis of oat hulls (experiment #7): (**a**) NOHs (×1000), (**b**) NOHs (×3000), (**c**) NOHs after hydrolysis (×1000), and (**d**) NOHs after hydrolysis (×3000).

Figure 1 displays the visualization of enzymatic hydrolysis of NOHs, specifically SEM images of NOHs and the residue after enzymatic hydrolysis with the enzyme cocktail consisting of the three preparations (experiment #7). The substrate before enzymatic hydrolysis (Figure 1a,b) had a lamellar, heterogeneous structure with surface cracks and roughness. The SEM image of the substrate after enzymatic hydrolysis (Figure 1c,d) shows that the enzymes acted on the surface of oat hulls and eliminated large irregularities. It is likely that the enzymes depolymerized all protrusions, smoothed out cracks and natural surface depressions, thereby leveling the surface without fragmenting it.

**Figure 2:**
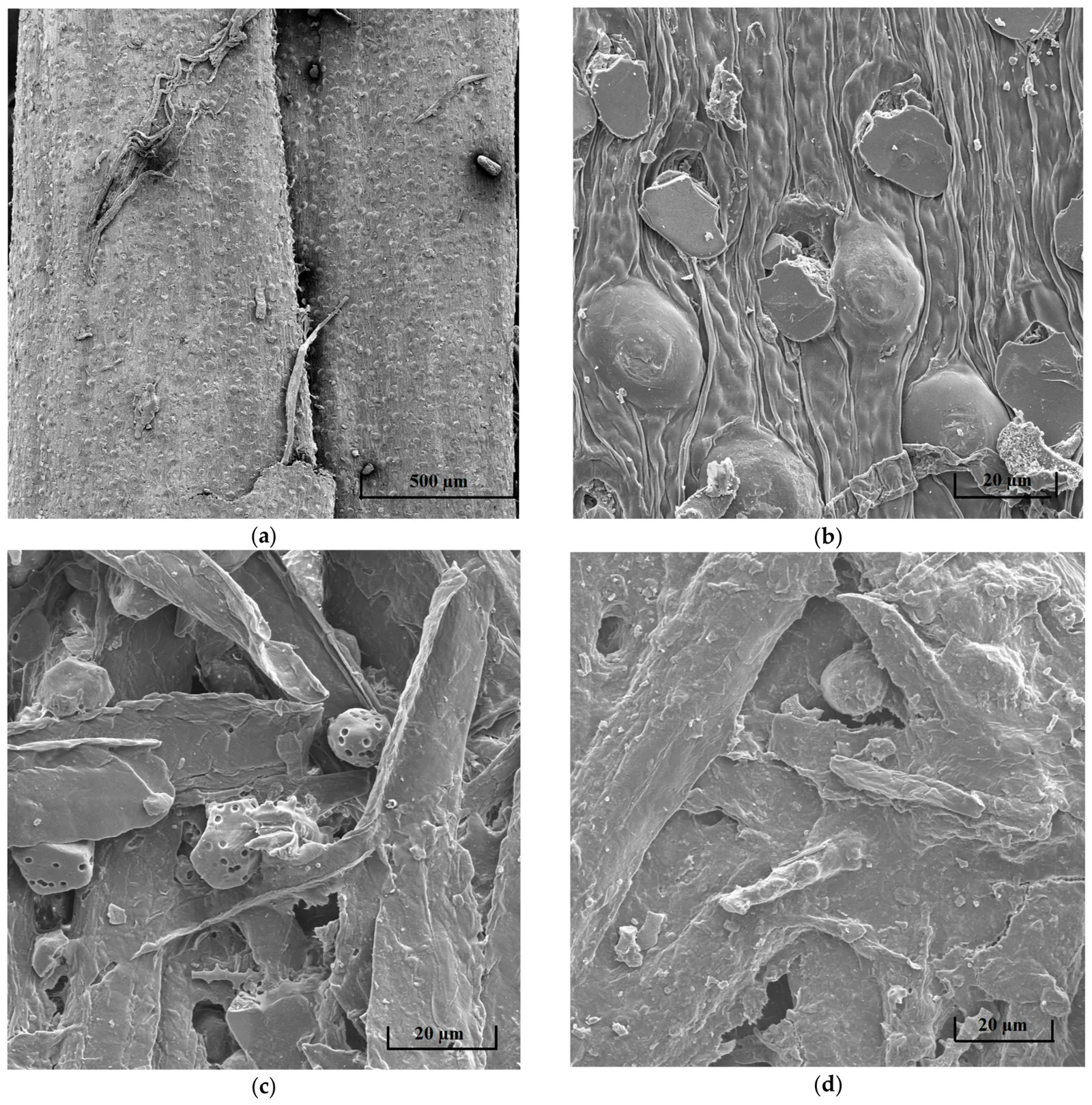

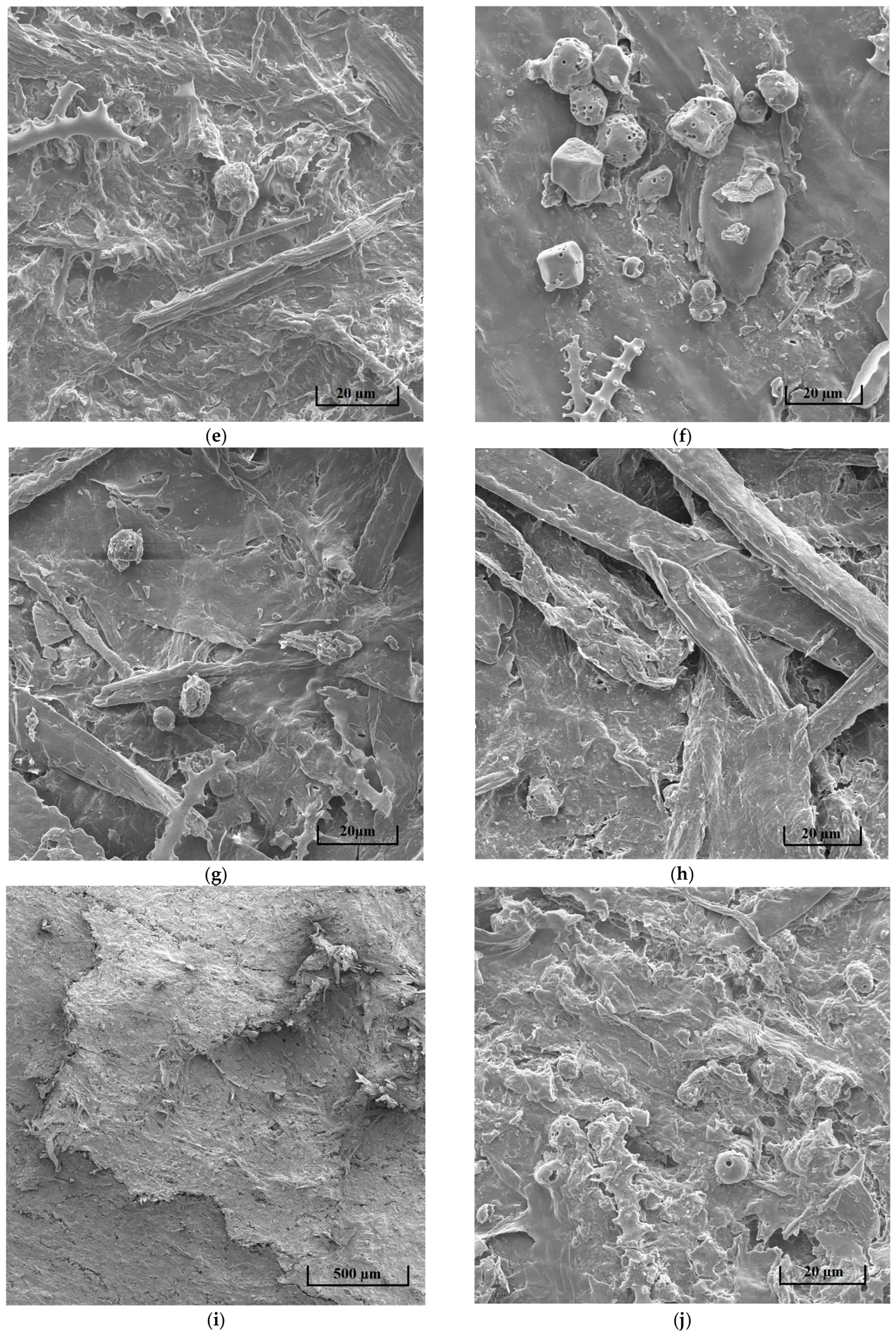
SEM visualization of enzymatic hydrolysis of oat hulls after pretreatment: (**a**) CPOHs (×100), (**b**) CPOHs (×2000), (**c**) CPOHs, Exp. 1 (×2000), (**d**) CPOHs, Exp. 2 (×2000), (**e**) CPOHs, Exp. 3 (×2000), (**f**) CPOHs, Exp. 4 (×2000), (**g**) CPOHs, Exp. 5 (×2000), (**h**) CPOHs, Exp. 6 (×2000), (**i**) CPOHs, Exp. 7 (×100), and (**j**) CPOHs, Exp. 7 (×2000).

The SEM results of the CPOH substrate before and after hydrolysis (Figure 2) provided remarkable insights into the transformations of lignocellulose upon enzyme attack, particularly when comparing the effects of individual enzymes and mixtures thereof. To the best of our knowledge, such results have not been reported in the literature.

At magnifications of ×100 and ×2000 (Figure 2a,b), the substrate consists of large flat fibers of varying sizes and fibrils with thicknesses ranging from 0.02 to 0.20 μm. The large fibers have an uneven surface, on which loop-like areas, cracks, pores, and regularly shaped protrusions can be observed. It can be assumed that the protrusions are silica formations that appeared during the nitric-acid treatment of oat hulls. The shape of the fibers arises from the specific morphology of thin lignocellulose scales that cover the grain in multiple layers to protect it from atmospheric precipitation. The unique fiber morphology of the substrate makes it difficult to compare the obtained SEM results with published data, as no such data are currently available. The surface heterogeneity, specifically the presence of roughness and pores on the fibers, is known to facilitate faster penetration of cellulases into the substrate [34].

After hydrolysis with Agrocell Plus (Figure 2c, experiment #1), the intact CPOH substrate fragmented into smaller, flat, ribbon-like fibers with uneven edges, measuring 10–40 μm in width and 200–700 μm in length. Fragmentation of the substrate particles was accompanied by the release of silica formations as irregularly shaped spheres of 10–20 μm in diameter and as comb-like structures with jagged edges 2–10 μm wide and up to 100 μm long. The emergence of silica formations is explained by the accumulation of lignin and ash in the solid phase, since cellulose and hemicelluloses are transferred to the liquid phase during hydrolysis as glucose, cellobiose, and soluble oligosaccharides. Similar phenomena of visualization of the enzymatic hydrolysis process, specifically the release of individual particles from the intact substrate upon enzyme attack, have been described in [35,36].

In contrast to experiment #1, the residue of the CPOH substrate after hydrolysis with Cellolux-A (Figure 2d, experiment #2) fragmented to a lesser extent, since larger fibers with a hypertrophied surface were observed following the enzyme attack only on the substrate surface. The presence of ribbon-like fibers and small particles of two distinct forms (irregularly shaped spheres and comb-like structures) confirms the superficial nature of the enzyme action. After hydrolysis with Ultraflo Max (Figure 2e, experiment #3), the substrate underwent more extensive fragmentation to generate very fine particles among which fibers of various lengths and shapes (tubes also being present) could be identified, albeit with difficulty, lying in a dense layer. Comparison of the SEM images of the substrate residues after hydrolysis with the individual enzyme preparations reveals different degrees of enzyme action, with the results of experiment #2 being inferior to those of experiments #1 and 3.

Hydrolysis with the cocktail of Agrocell Plus and Ultraflo Max (Figure 2f, experiment #4) transformed the CPOH substrate into a diversity of particle forms, among which flat twisted ribbons, tubes, and spherical and comb-like silica formations were present in approximately equal proportions, confirming the most extensive fragmentation among the experiments described above. It should be emphasized that the presence of Ultraflo Max in the mixture, similarly to experiment #3, led to the formation of tubular fibers in the CPOH substrate residue, and holes in the flat fibers and significant deformation of the edges of these fibers were also observed for the first time.

Compared with the previous experiment, mixed Agrocell Plus/Cellolux-A (Figure 2g, experiment #5) acted much more weakly on the CPOH substrate, as only large fibers were observed in the residue; however, the particle size indicates more efficient fragmentation than in the experiments with individual enzyme preparations. A significant improvement compared to experiment #5 was observed when the CPOH substrate was treated with mixed Cellolux-A/Ultraflo Max (Figure 2h, experiment #6), as the residual particles were difficult to identify and they formed a dense layer of a dried-out suspension.

The use of the three-enzyme mixture (Figure 2i,j, experiment #7) led to almost complete hydrolysis of the accessible fraction of the CPOH substrate, resulting in a homogeneous amorphous mass of very fine particles of various shapes, among which irregularly shaped spheres and comb-like structures characteristic of silica formations could be observed upon magnification. This is to be expected, since the mineral components, which are not hydrolyzed, must accumulate in the solid residue during enzymatic hydrolysis. The comparison of the comb-like structures (Figure 2i,j, experiment #7) with those published for biogenic silica from oat hulls [15] demonstrates complete similarity, confirming our assumptions. Thus, the idea of obtaining biogenic silica through enzymatic hydrolysis of cellulosic biomass followed by incineration of the substrate residue remains highly relevant [17]. Since the value of biogenic silica has been repeatedly demonstrated [37,38], the use of multienzyme mixtures in the hydrolysis of CPOHs allows the production of one more valuable product, amorphous silicon dioxide (silica), in addition to a glucose hydrolyzate with a concentration of 22 g/L.

Summarizing the results of CPOH hydrolysis visualization, it should be noted that the substrate residues after hydrolysis consisted of a disordered mixture of tiny fiber fragments and silica formations in most cases. When individual enzyme preparations were used, larger fiber fragments were present in the residues, whereas the residue consisted of an amorphous mass comprising only small fiber fragments and silica formations when enzyme cocktails were used, thereby confirming the effective use of enzyme cocktails. It should be noted that the described silicon formations (irregularly shaped spheres and comb-like structures) are observed only in the SEM images of oat hulls after the pretreatment and subsequent enzymatic hydrolysis (Figure 2c–j). The findings obtained in this study are in good agreement with the high RS yields achieved in experiments #4, 6, and 7 (Table 2).

The analysis of the published data on the visualization of enzymatic hydrolysis shows scarce examples of how enzyme cocktails act on substrates. The study [19] reported the visualization of enzymatic hydrolysis of sweet sorghum straw before and after pretreatment and revealed that cellulase dissolved the cell walls in the same manner, and the overall digestibility was negatively correlated with the lignin content of the substrate. It was also noted [19] that a deeper understanding of the processes of pretreatment and enzymatic hydrolysis of biomass is the key to lignocellulose conversion.

A logical continuation of this research is the scale-up of enzymatic hydrolysis and the use of higher initial solid loadings, since the RS yield is known to be inversely related to the substrate loading [25]. Apart from glucose hydrolyzates, the possibility of obtaining other valuable products, such as amorphous silicon dioxide, may justify a reduction in the yield of target glucose when using a highly efficient multienzyme mixture in industrial biocatalysis.

### 2.4. On the Issue of Mass Balance and Scale-Up

#### 2.4.1. Mass Balance

**Figure 3:**
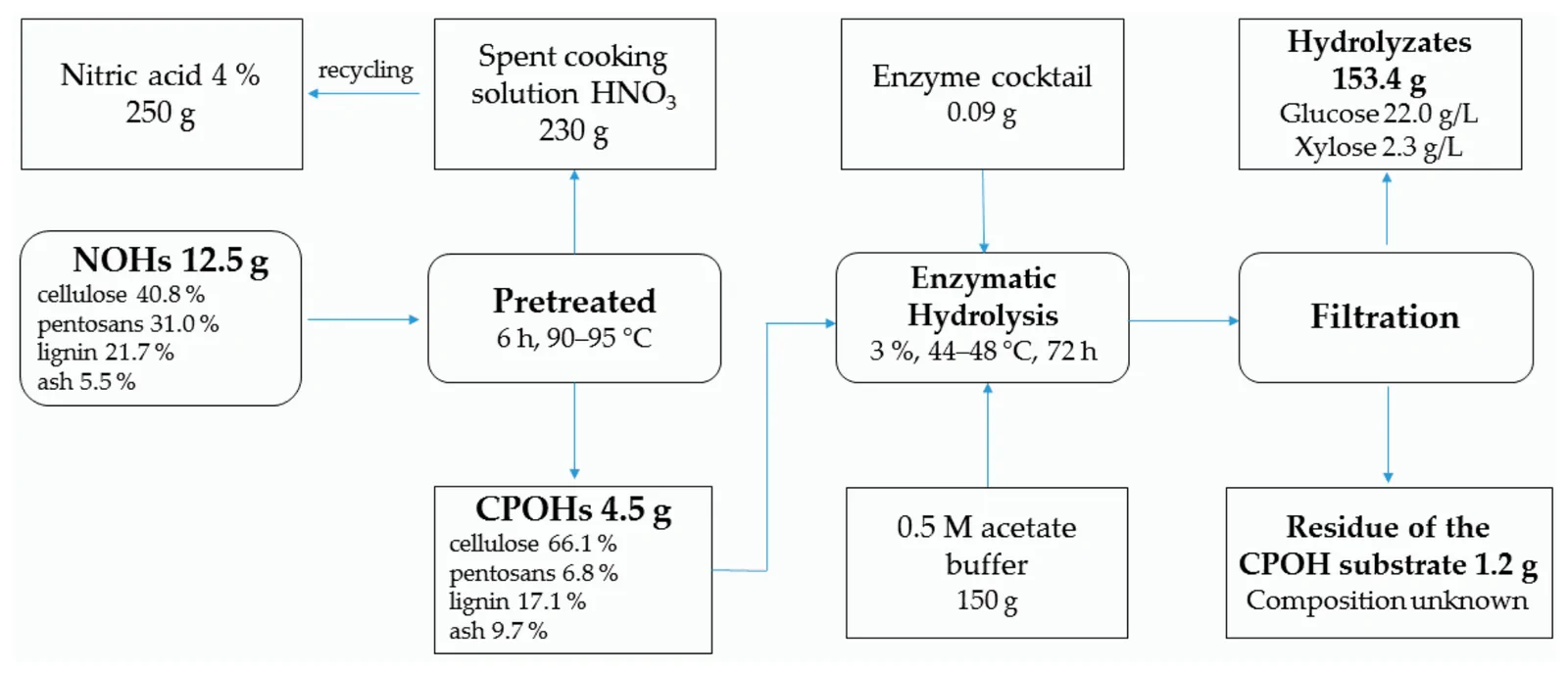
Mass balance of the pretreatment of oat hulls (NOHs) and the enzymatic hydrolysis of the pretreatment product (CPOHs).

Figure 3 presents the mass balance of the pretreatment of oat hulls (NOHs) and the enzymatic hydrolysis of the pretreatment product (CPOHs) carried out in this work.

Treating the feedstock with a dilute nitric acid solution is quite a technologically efficient and environmentally friendly method, as this process is carried out at atmospheric pressure, uses a low acid concentration of 4%, and requires the use of hermetically sealed equipment for the capture of nitrogen oxides, which are returned to the cooking liquor and play an important role in this treatment [39]. The yield of CPOHs from the feedstock is 36%; in other words, a major portion (64%) of the solid phase of the feedstock is solubilized in the cooking liquor, resulting in 4.5 g of the CPOH substrate from 12.5 g of the feedstock. An advantage of this method is the cooking liquor can be recycled more than five times, as demonstrated in [39]. Moreover, the spent cooking liquor is neutralized with ammonia to produce fertilizers [29]. The compositions of the feedstock and the pretreatment product presented in this work (Table 1, Figure 3) allow for the conclusions that the product is enriched with cellulose (62%) and ash (76%) due to the removal of most of pentosans (78%) and some of lignin (21%) from the feedstock.

The enzymatic hydrolysis of 4.5 g of the CPOH substrate in 150 g of acetate buffer resulted in 153.4 g of the target hydrolyzate after filtration, in which 3.4 g of glucose and 0.35 g of xylose are dissolved, along with 1.21 g of the solid phase residue whose composition has not yet been studied, but the SEM results show particles of various shapes that correspond to biogenic silica by appearance [15]. The target hydrolyzate can be used as a component of a nutrient medium in the biosynthesis of bacterial nanocellulose, as reported in Section 3.2. Thus, 153.4 g of a predominantly glucose hydrolyzate (containing 3.4 g of glucose), 1.21 g of a biogenic silica precursor, and 230 g of a byproduct of the solution can be obtained from 12.5 g of oat hulls. After neutralization, the solution can be used as a fertilizer [29].

#### 2.4.2. Scale-Up

In this work, a relatively low substrate loading of 3% was used. This choice is justified based on publications by other authors, who typically use a substrate concentration of 2–5% for studying the enzyme–substrate interactions [40]. The glucose concentration in the synthetic Hestrin–Schramm nutrient medium is known to be 20 g/L for effective cultivation of bacterial nanocellulose [41]. To scale up the cultivation of bacterial nanocellulose and refuse from the synthetic nutrient medium, the possibility of switching to enzymatic hydrolyzates derived from cellulosic raw materials is being discussed [42,43,44,45]; therefore, the researchers use glucose concentrations not exceeding 20 g/L and compare the obtained results with the yield and properties of bacterial nanocellulose in Hestrin–Schramm experiments at 20 g/L [41]. For example, Liu et al. [45] discussed successful biosynthesis of bacterial nanocellulose on three enzymatic hydrolyzates (vinegar residue, paper mulberry, and seagrass) with glucose concentrations of 14.78, 11.73, and 12.23 g/L, which are lower than our result of 22 g/L. Liu et al. [45] employed a method for determining the concentration of reducing sugars, which proved itself in practice [46], compared the obtained results with biosynthesis on the Hestrin–Schramm nutrient medium with a 20 g/L glucose concentration, and believed that the technical solutions they proposed can be implemented on an industrial scale.

Therefore, the enzymatic hydrolyzate derived from CPOHs in our work, with a glucose concentration of 22 g/L, can be used as a nutrient medium for the production of a specific biosynthetic product on an industrial scale. According to reviews on scaling up the bacterial nanocellulose biosynthesis [42,43,44], the biotransformation of agro-industrial waste into high-value bio-based products can create innovative sources of income, especially for the agricultural and food industries. Also, it is precisely this model of resource efficiency that will reduce reliance on synthetic feedstocks in the long term. For the preparation of nutrient media for microbiological synthesis, e.g., ethanol, it is necessary to run the process at elevated initial substrate concentrations of 10–20%, which represents a logical continuation of our research. The RS yield is known to be in inverse relation to the initial substrate concentration [20,21,40]. Operating factories encounter problems when running the process at elevated substrate concentrations of >10%, associated with the enzyme–substrate interactions and inhibition by the final product. This problem can be solved through the use of highly efficient cellulase preparations for hydrolysis [40]. The enzyme cocktail composed of three enzyme preparations used in our work demonstrated its high efficiency (99% hydrolysis of the available part of the substrate), which allows for the assumption that, although the RS yield will decrease as the substrate loading is raised, it will nevertheless achieve high RS concentrations compared with individual enzymes. We are planning on a study of enzymatic hydrolysis at initial substrate concentrations of 10–20% or with fed-batch operation.

## 3. Materials and Methods

### 3.1. Feedstock and Pretreatment

Oat hulls (*Avena sativa*) provided by Diarit LLC (Tambov, Russia) were used as the feedstock in this study. Pretreatment of oat hulls was carried out with a 4% nitric acid solution (CAS No. 7697-37-2, 99.0%, Chimmedsnab, Korolev, Russia) for 6 h at 90–95 °C. The product obtained after nitric-acid treatment was washed with water until neutral pH, then air-dried for compositional analysis and subsequent enzymatic hydrolysis. As a result, the following two types of substrates were studied: native oat hulls (NOHs) and chemically pretreated oat hulls (CPOHs).

### 3.2. Enzyme Preparations

The following three enzyme preparations acquired from different manufacturers were used for hydrolysis: Agrocell Plus (Agroferment LLC, Moscow, Russia), CelloLux-A (Sibbiofarm LLC, Novosibirsk, Russia), and Ultraflo Max (Novozymes A/S, Bagsværd, Denmark). Agrocell Plus and CelloLux-A are described by the manufacturers as multienzyme preparations that exhibit cellulase, xylanase, and β-glucanase activities. Ultraflo Max is a binary mixture of β-glucanase and xylanase. The microbial producers and enzymatic activities of the preparations are outlined in Table 4.

**Table 4:** Microbial producers and enzymatic activities of the enzyme preparations.

Enzyme	Microbial Producer	Enzymatic Activity, U/g
Agrocell Plus	*Penicillium verruculosum*	Cellulase, 4000 β-Glucanase, 3000 Xylanase, 400
CelloLux-A	*Trichoderma viride* (*reesei*)	Cellulase, 2000 β-Glucanase, 1500 Xylanase, 6000
Ultraflo Max	*Aspergillus oryzae* *Trichoderma reesei*	β-Glucanase, 700 Xylanase, 250

### 3.3. Substrate Analysis

The moisture content of the substrates was determined using an MB23 moisture analyzer (Ohaus, Parsippany, NJ, USA). Compositional analysis of the substrates was carried out according to standard methods for plant biomass analysis. The cellulose content was determined by the Kürschner method via extraction with mixed HNO_3_/ethanol (1:4, *v*/*v*) for 4 h [47]. The acid-insoluble lignin content was evaluated according to the method described in [48]. The pentosan content was determined using an orcinol solution (CAS No. 106-50-3, Khimleader LLC, Barnaul, Russia) spectrophotometrically on a UNICO UV-2804 spectrophotometer (United Products and Instruments, Dayton, NJ, USA) [30]. Ash content was determined by incinerating the sample at 600 °C for 3 h [49].

The morphology of the substrates and unreacted residues after hydrolysis was studied by scanning electron microscopy (SEM) (Jeol Ltd., Tokyo, Japan) [50].

### 3.4. Enzymatic Hydrolysis

The hydrolytic properties of the enzyme preparations were evaluated under identical conditions as follows: a solid loading of 3% and a stirring speed of 150 rpm on an EKROS PE-6410 horizontal mixing device (Ecokhim, Moscow, Russia) at 46 ± 2 °C. The substrate weighing 4.5 g an oven-dry basis was placed into a 0.5 L Erlenmeyer flask. The total reaction volume of 0.15 L was maintained by adding 0.5 M acetate buffer (pH 4.7). For bioconversion, the enzymes were used individually (experiments 1–3) and in combinations (enzyme cocktails) whose formulations are presented in Table 5 (experiments 4–7). The enzyme preparations were mixed in a 1:1 (or 1:1:1) ratio in all possible combinations. Prior to addition, the individual enzyme preparations or their cocktails were dissolved in an acetate buffer. The process was run at an enzyme loading of 20 mg of enzyme per gram of substrate or 0.6 mg of enzyme per mL of reaction mixture. The experimental results were obtained in triplicate and statistically processed by one-way analysis of variance (ANOVA). The results of the one-way ANOVA showed statistically significant differences across the groups (*p* < 0.05).

After 72 h hydrolysis, the suspension was vacuum-filtered, and the filtrate was analyzed for RS, glucose, and pentose concentrations. The concentration of reducing sugars (RS) was quantified on a glucose basis using a 3,5-dinitrosalicylic acid reagent (Panreac, Castellar del Vallès, Spain) [46] on a Cary 60 UV-Vis spectrophotometer (Agilent Technologies, Santa Clara, CA, USA). The glucose concentration was quantified by the glucose oxidase-peroxidase method using reagents from the Fotoglyukosa kit (Impact LLC, Moscow, Russia) [51]. The xylose concentration was measured chromatographically according to the procedure detailed in [31]. HPLC analysis of xylose in the hydrolyzates was performed on a Milichrom A-02 microcolumn liquid chromatograph (EcoNova, Novosibirsk, Russia) using a Ø 2 × 75 mm column filled with ProntoSIL-120-5-c18 sorbent (Bischoff Analysentechnik und -geräte GmbH, Leonberg, Germany). This method is based on the derivatization reaction of hydrolyzate monosugars with 2,4-dinitrophenylhydrazine. Chromatographic conditions were as follows: 0.1% trifluoroacetic acid as eluent A and 0.1% trifluoroacetic acid and 70% acetonitrile as eluent B; gradient 2200 μL 16–27% B, 200 μL 27–100% B, and 600 μL 100% B; eluent flow rate of 150 μL/min, temperature 35 °C; 360 nm wavelength for detection; and sample volume of 5 μL. Based on the analysis results, the final yields of RS (Equation (1)) on a substrate weight basis and (Equation (2)) on a hydrolyzables basis, as well as the glucose yield (Equation (1)) on a substrate weight basis and (Equation (3)) on a cellulose content basis, were calculated by the equations:

(1) $$\eta_{S}=\frac{C_{F}\cdot V}{m_{S}}\cdot 0.9\cdot 100,$$

(2) $$\eta_{HC}=\frac{C_{F}\cdot V}{m_{S}\cdot (C+P)/100\;}\cdot 0.9\cdot 100,$$

(3) $$\eta_{C}=\frac{C_{F}\cdot V}{m_{S}\cdot C/100}\cdot 0.9\cdot 100.$$

where *η_S_* is the RS yield (glucose) on a substrate weight basis, %;

*η_HC_* is the RS yield relative to the hydrolyzable content of the substrate, %;

*η_C_* is the glucose yield relative to the cellulose content of the substrate, %;

*C_F_* is the final RS concentration in the hydrolyzate, g/L;

*V* is the hydrolyzate volume, L;

0.90 is the coefficient attributed to the water molecule addition to anhydroglucose residues of the corresponding monomer units as a result of enzymatic hydrolysis;

*m_S_* is the substrate weight for enzymatic hydrolysis, g;

*C* is the mass content of cellulose in the substrate, %.

*P* is the mass content of pentosans in the substrate, %.

## 4. Conclusions

This study on the effect of enzyme preparations and their cocktails on the hydrolysis efficiency of native oat hulls (NOHs) and chemically pretreated oat hulls (CPOHs) revealed that the use of an enzyme cocktail consisting of three enzyme preparations of different origin (Agrocell Plus, Cellolux-A, and Ultraflo Max) leads to a significant increase in the substrate conversion degree. In the case of NOHs, the reducing sugar (RS) yield on a hydrolyzables content basis was 63%, while the yield of 99% was achieved for CPOHs. Such a high hydrolysis efficiency of the CPOH substrate can be attributed to two factors. First, it is associated with the pretreatment of NOH, which increases the susceptibility of the hydrolyzable components of the feedstock to enzymatic hydrolysis. Second, the use of a highly efficient enzyme cocktail consisting of enzyme preparations of different origin significantly improved the hydrolysis in this case.

SEM visualization of enzymatic hydrolysis revealed differences in the mechanism of enzyme attack on the feedstock before and after pretreatment as follows: in the case of NOHs, hydrolysis occurred on the substrate surface, whereas hydrolysis led to substrate disintegration in the case of CPOHs. The SEM results visualize the advantage of using enzyme cocktails over individual enzymes.

CPOH hydrolyzates were found to be chiefly glucose-rich, with glucose accounting for 91% of the total RS, indicating the significant potential of using this substrate in the processes of enzymatic hydrolysis and subsequent biosynthesis, including those at higher initial solid loadings.

The mass balance assessment showed that 12.3 kg of a predominantly glucose hydrolyzate containing 0.27 kg of glucose and 0.03 kg of xylose, 0.96 kg of a biogenic silica precursor, and 18.4 kg of a byproduct of the solution can be obtained from 1.0 kg of oat hulls. Following neutralization, the solution can be utilized as a fertilizer.

The results obtained in this work using the enzyme cocktail consisting of three enzyme agents (99% hydrolysis of the available part of the substrate) allow us to assume its high efficiency for scaling up the process with an increase in the initial solid loading.

Thus, NOHs represent a promising type of lignocellulose for facilitating the transition to sustainable processing of grain waste to establish enzymatic hydrolysis with subsequent fermentation as a cornerstone technology that reduces waste and creates economically demanded products.

## Figures and Tables

**Table 1:** Composition of native oat hulls (NOHs) and chemically pretreated oat hulls (CPOHs).

Components	Oat Hulls (NOHs)	Oat Hulls After Pretreatment (CPOHs)
Mass content of cellulose, %	40.8 ± 0.4	66.1 ± 0.4
Mass content of pentosans, %	31.0 ± 0.1	6.8 ± 0.1
Mass content of acid-insoluble lignin, %	21.7 ± 0.1	17.1 ± 0.1
Mass content of ash, %	5.5 ± 0.01	9.7 ± 0.01

Data are presented as mean ± SD.

**Table 2:** Characteristics of hydrolyzates derived from NOHs after 72 h hydrolysis.

Hydrolyzate Characteristics	Experiment #						
	1	2	3	4	5	6	7
**RS:**							
Concentration, g/L	6.6 ± 0.5	3.9 ± 0.5	3.1 ± 0.5	7.3 ± 0.5	4.7 ± 0.5	11.2 ± 0.5	15.0 ± 0.5
Yield on a substrate weight basis, %	20 ± 2	12 ± 2	9 ± 2	22 ± 2	14 ± 2	34 ± 2	45 ± 2
Yield on a hydrolyzables basis, %	28 ± 2	17 ± 2	13 ± 2	31 ± 2	20 ± 2	47 ± 2	63 ± 2
**Glucose:**							
Concentration, g/L	3.0 ± 0.8	1.6 ± 0.8	1.5 ± 0.8	3.2 ± 0.8	2.0 ± 0.8	5.0 ± 0.8	6.1 ± 0.8
Yield on a substrate weight basis, %	9 ± 3	5 ± 3	5 ± 3	10 ± 3	6 ± 3	15 ± 3	18 ± 3
Yield on a cellulose content basis, %	22 ± 3	12 ± 3	11 ± 3	24 ± 3	15 ± 3	37 ± 3	45 ± 3
Xylose concentration, g/L	3.0 ± 0.2	2.0 ± 0.2	1.4 ± 0.2	4.0 ± 0.2	2.4 ± 0.2	5.6 ± 0.2	8.5 ± 0.2

Data are presented as mean ± SD.

**Table 3:** Characteristics of hydrolyzates from pretreated oat hulls after 72 h of hydrolysis.

Characteristics of Hydrolyzates	Experiment #						
	1	2	3	4	5	6	7
**RS:**							
Concentration, g/L	17.6 ± 0.5	13.2 ± 0.5	16.9 ± 0.5	23.7 ± 0.5	19.3 ± 0.5	23.6 ± 0.5	24.1 ± 0.5
Yield on a substrate weight basis, %	53 ± 2	40 ± 2	51 ± 2	71 ± 2	58 ± 2	71 ± 2	72 ± 2
Yield on a hydrolyzables basis, %	72 ± 2	54 ± 2	69 ± 2	97 ± 2	79 ± 2	97 ± 2	99 ± 2
**Glucose:**							
Concentration, g/L	15.5 ± 0.8	11.9 ± 0.8	14.4 ± 0.8	21.2 ± 0.8	16.9 ± 0.8	20.9 ± 0.8	22.0 ± 0.8
Yield on a substrate weight basis, %	46 ± 3	36 ± 3	43 ± 3	64 ± 3	51 ± 3	63 ± 3	66 ± 3
Yield on a cellulose content basis, %	70 ± 3	54 ± 3	65 ± 3	96 ± 3	77 ± 3	95 ± 3	100 ± 3
Xylose concentration, g/L	1.9 ± 0.2	1.7 ± 0.2	2.0 ± 0.2	2.3 ± 0.2	1.7 ± 0.2	2.2 ± 0.2	2.3 ± 0.2

Data are presented as mean ± SD.

**Table 5:** Experimental design: enzyme preparations used in each experiment.

Experiment #	Enzyme Preparations Used		
	Agrocell Plus	CelloLux-A	Ultraflo Max
1	+		
2		+	
3			+
4	+		+
5		+	+
6	+	+	
7	+	+	+

## Data Availability

The data that support the findings of this study are contained within the article.
